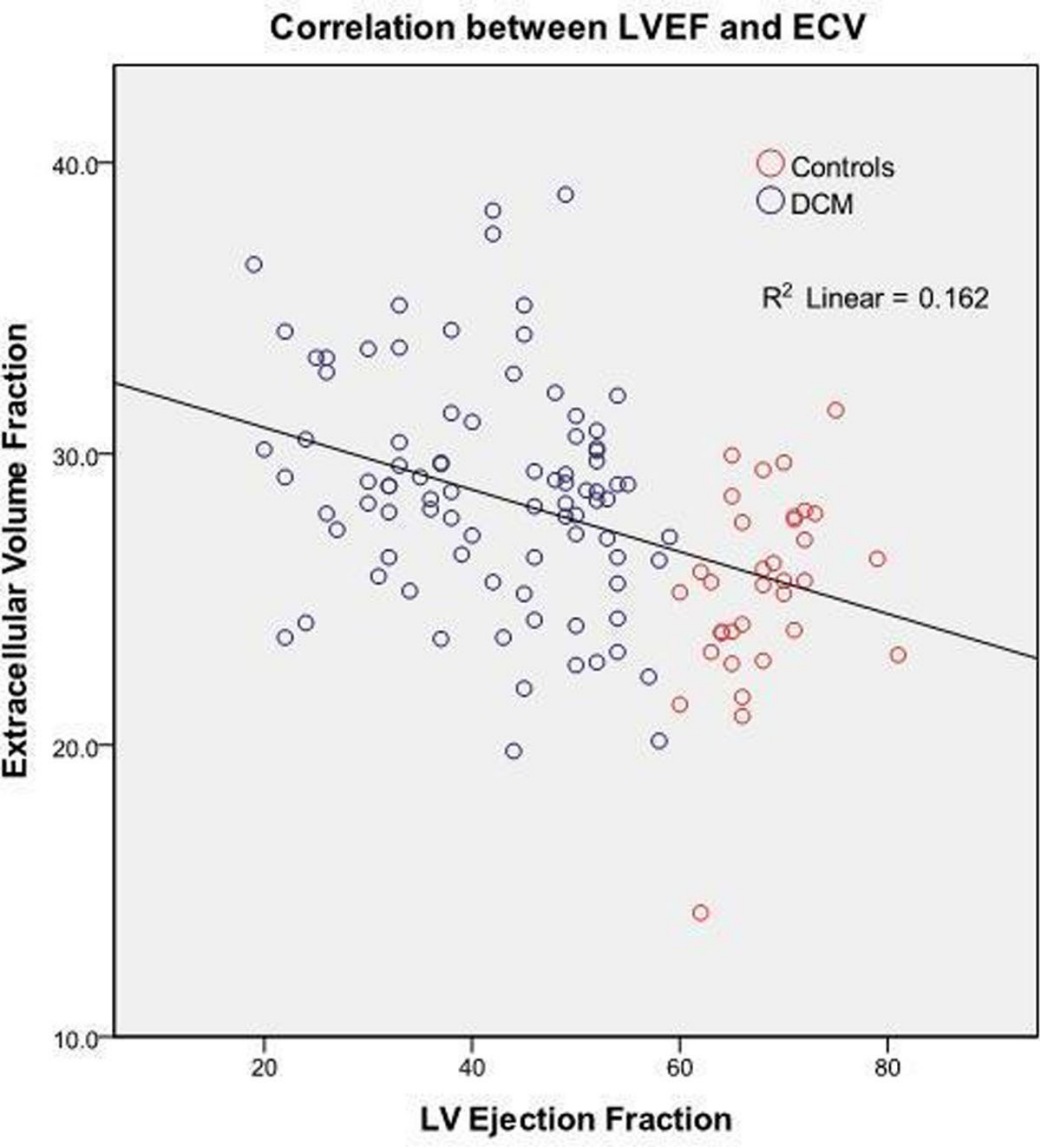# Quantitative assessment of myocardial extracellular volume fraction in non-ischemic dilated cardiomyopathy and its relation to systolic dysfunction

**DOI:** 10.1186/1532-429X-15-S1-O19

**Published:** 2013-01-30

**Authors:** Aamir Ali, Ankur Gulati, Tevfik F Ismail, Kaushiga Krishnathansan, Nizar Ismail, Evangelia Nyktari, Navtej Chahal, Claire E Raphael, Rick Wage, Taigang He, Peter D Gatehouse, David Firmin, Peter Kellman, Dudley Pennell, Andrew E Arai, Sanjay K Prasad

**Affiliations:** 1 CMR Unit, Royal Brompton Hospital, London, UK; 2 NHLBI, National Institutes of Health, Bethesda, MD, USA

## Background

Interstitial myocardial fibrosis is a histological hallmark of non-ischemic dilated cardiomyopathy (DCM), and may play an important role in adverse remodelling and progressive systolic dysfunction. T1-mapping enables non-invasive assessment of diffuse fibrosis by quantification of myocardial extracellular volume fraction (ECV). We hypothesized that CMR would identify a raised ECV in DCM which would correlate with the degree of systolic dysfunction.

## Methods

Consecutive DCM patients referred for CMR and age/sex-matched healthy controls were prospectively enrolled. Exclusion criteria included a history of recent myocarditis, ischemic heart disease, diabetes, severe hypertension and primary valvular disease. All subjects underwent CMR (1.5T, Siemens Avanto) according to a standardized protocol which included T1-mapping and late gadolinium enhancement (LGE) imaging. Mid-ventricular short-axis T1-maps were acquired using a Modified Look-Locker Inversion recovery sequence prior to contrast and 20 minutes after gadolinium administration (Gadovist 0.1mmol/kg). The pre- and post-contrast T1-maps were co-registered and used with the patient's hematocrit to generate an ECV map.

## Results

In total, 85 patients (58 male, mean age 51.5yrs, mean left ventricular ejection fraction [LVEF] 42%) and 35 controls (22 male, mean age 46yrs, mean LVEF 68%) were studied. Baseline clinical and CMR characteristics for the cohort are summarized in Table 1. Mid-wall LGE was present in 14 (16.5%) DCM patients. In one patient, LGE was observed in the same short-axis slice as the T1-map and this study was therefore excluded from analysis. Patients with DCM had significantly higher ECV compared to controls (28.8 ±3.9% vs. 25.5 ±3.2%, p < 0.0001). A significant negative correlation was observed between ECV and LVEF (r=-0.54, p< 0.0001). Univariate linear regression analysis revealed that indexed left ventricular end diastolic volume, heart rate, gender and LVEF were significantly associated with ECV. On multivariate analysis, only LVEF (B = -0.12, 95% CI -0.17 to -0.06, p<0.0001) and gender (B = -3.2, 95% CI -4.6 to -1.9, p < 0.0001) were independent determinants of ECV.

**Table 1:** Baseline clinical and CMR characteristics

Characteristic	Disease (n=85)	Control (n=35)	*p*-value
**Age (years)**	52	46	0.067
**Male**	58	22	0.629
**Heart rate (bpm)**	73	63	<0.0001
**Systolic BP (mmHg)**	122	120	0.698
**Diastolic BP (mmHg)**	74	76	0.559
**LV-EDVi (mL)**	133	81	<0.0001
**LV-ESVi (mL)**	80	26	<0.0001
**LVEF**	41	68	<0.0001
**LVMI (g/m^2^)**	82	57	<0.0001

Beats per minute (bpm); blood pressure (BP); indexed left ventricular end-diastolic colume (LV-EDVi); indexed left ventricular end-systolic volume (LV-ESVi); left ventricular ejection fraction (LVEF); indexed left ventricular mass (LVMI)

## Conclusions

ECV is expanded in DCM in proportion to the degree of LV systolic dysfunction. An increased ECV in sections of the heart without clinically obvious LGE suggests the presence of low level myocardial fibrosis or possibly myocardial edema. This technique offers potential for the evaluation of interstitial fibrosis in DCM, although important challenges include the substantial overlap of ECV values between patients and normals. Further work should aim to corroborate these findings with histological validation.

## Funding

This project was supported by the NIHR Cardiovascular Biomedical Research Unit of Royal Brompton and Harefield NHS Foundation Trust, the British Heart Foundation, and CORDA (research charity).

Dr Peter Kellman and Dr Andrew Arai are funded by the National Heart, Lung and Blood Institute, NIH, Bethesda, MD, USA.

**Figure 1:**